# Comparison of central composite design and factorial arrangement to evaluate the interaction between net energy, soybean meal, and standardized ileal digestible lysine content of diets fed to pigs from 11 to 25 kilograms

**DOI:** 10.1093/jas/skaf316

**Published:** 2025-09-13

**Authors:** Hilario M Cordoba, Mike D Tokach, Jason C Woodworth, Katelyn N Gaffield, Robert D Goodband, Joel M DeRouchey, Jordan T Gebhardt, Henrique S Cemin, Jose A Soto

**Affiliations:** Department of Animal Sciences and Industry, College of Agriculture, Kansas State University, Manhattan, KS, USA; Department of Animal Sciences and Industry, College of Agriculture, Kansas State University, Manhattan, KS, USA; Department of Animal Sciences and Industry, College of Agriculture, Kansas State University, Manhattan, KS, USA; Department of Animal Sciences and Industry, College of Agriculture, Kansas State University, Manhattan, KS, USA; Department of Animal Sciences and Industry, College of Agriculture, Kansas State University, Manhattan, KS, USA; Department of Animal Sciences and Industry, College of Agriculture, Kansas State University, Manhattan, KS, USA; Department of Diagnostic Medicine/Pathobiology, College of Veterinary Medicine, Kansas State University, Manhattan, KS, USA; Hubbard Feeds, Mankato, MN, USA; Alltech, Nicholasville, KY, USA

**Keywords:** central composite design, factorial arrangement, lysine, net energy, nursery pigs, soybean meal

## Abstract

Two studies compared a central composite design (CCD) and a factorial arrangement of treatments to evaluate the effects of dietary net energy (NE), soybean meal (SBM), and standardized ileal digestible (SID) Lys on the growth performance of 11 to 25 kg pigs. Experiment 1 used 4,681 pigs (PIC 337 × 1,050; initially 13.0 kg) in a CCD with approximately 35 pigs per pen and seven blocks, each comprising 19 pens: eight factorial points, six axial points, and a central point replicated five times. Eight diets were formulated to various NE, SBM, and SID Lys concentrations then blended to create the 15 dietary treatments. Net energy ranged from 2,334 to 2,762 kcal/kg, SBM from 25.5% to 35.9%, and SID Lys from 1.08% to 1.52%. Increasing SID Lys quadratically increased (*P *< 0.05) ADG and G:F. Increasing SBM linearly increased (*P *< 0.05) ADG and G:F. Increasing NE decreased (linear, *P *< 0.10) ADG due to a reduction in the Lys:NE ratio as NE increased. An SBM × SID Lys interaction (*P *= 0.082) was observed for G:F, where SID Lys increased G:F with increasing SBM due to decreasing the Lys:CP ratio. In experiment 2, there were 4,336 pigs (PIC 337 × 1,050; initially 10.6 kg) with approximately 34 pigs per pen and eight pens per treatment arranged in a 2 × 2 × 4 factorial. Eight diets with various NE, SBM, and SID Lys levels were blended to create the 16 dietary treatments. Main effects included NE (2,425 or 2,676 kcal/kg), SBM (25.5% or 33.5% of the diet), and SID Lys (1.08%, 1.20%, 1.31%, or 1.43%). There was a tendency (*P *= 0.063) for three-way interaction for ADG driven by the linear increase (*P *< 0.001) in ADG as SID Lys increased in diets containing 2,627 kcal NE/kg and 33.5% SBM compared to diets at lower SBM and NE with a higher Lys:NE ratio. A three-way interaction (linear, *P *= 0.023) was observed for G:F. Increasing SBM increased G:F to a greater extent in low-energy diets than in high-energy diets. Increasing SID Lys resulted in a greater response in high-energy diets than in low-energy diets. Diets containing low SID Lys and NE but high SBM increased G:F compared with low SID Lys, NE, and SBM also contributing to the interaction. Data from experiment 1 predicted results for almost all variables from experiment 2 within ±3% of the observed values. In conclusion, a CCD can provide similar estimates of pig growth performance as a factorial arrangement. In addition, both experiments showed the impact of not maintaining Lys:NE ratios when increasing NE and the benefit in G:F when increasing SBM in the diet.

## Introduction

The central composite design (CCD) permits the estimation of first- and second-order terms and quantifies the relationship between variables. Box and Wilson (1951) were the first to report this design, which allows the opportunity to explore the relationship between two or more variables with more than three levels. The central, factorial, and axial points represent the experimental domain that establishes the low and high extreme values for treatment variables (Bhattacharya, 2021). The CCD provides a three-dimensional analysis with more uniformity and precision in defining how the response surface behaves around the point of optimum conditions compared to the factorial arrangement (Penneton et al., 1999). Also, it can have the advantage of reducing the number of treatments needed compared to a three-way factorial experiment to cover the desired ranges for all three factors.

The CCD has been widely used in poultry research since the 1960s (Yoshida et al., 1962). Humphrey et al. (2023) recently used a CCD design to evaluate the interaction of branched-chain amino acids in nursery pig diets; however, the CCD model has been less frequently applied in swine compared to poultry research. Although a CCD reduces the number of treatments compared to a full factorial experiment, it still requires a large number of experimental units, often limiting its application in swine research.

Alternatively, a factorial arrangement consists of two or more variables with different levels of each that are compared within every other variable in the experiment. The factorial arrangement requires a greater number of treatments than a CCD to evaluate interactions and main effects (Aaron and Hays, 2001). This treatment arrangement has been widely used in swine nutrition research to investigate the interactions and main effects of two variables of interest. However, when evaluating multiple variables, factorial arrangements can be limited by the number of replications depending on the number of experimental units (pens) available for use.

From 11 to 25 kg, pigs experience a period of rapid growth and increased G:F. Pig growth responses to variable net energy (NE) concentrations have been demonstrated to be inconsistent and relative to the ingredients used in formulation (Oresanya et al., 2007; De Jong et al., 2014; Lunedo et al., 2023). In addition, maintaining the standardized ileal digestible lysine (SID Lys) to calorie (Lys:NE) ratio has been shown to improve growth performance (Marçal et al., 2019), but a recent review also reported that extra growth could be captured when increasing SID Lys over the optimum Lys:NE ratio (Goethals et al., 2024). Soybean meal (SBM) is commonly used in swine diets because it provides an excellent profile of essential and non-essential amino acids (Pope et al., 2023). Previous research has observed variable growth performance responses when feeding up to 40% SBM to pigs from 11 to 25 kg (Cemin et al., 2020a; Faccin et al., 2024). The response to increasing SID Lys, NE, or SBM individually, or with two of the three variables has been tested, but no research has evaluated the response surface to changing all three variables at the same time.

Therefore, the objective of these trials was to determine the effect of diets differing in NE, SBM, and SID Lys on the growth performance of late nursery pigs using both a factorial arrangement and a central composite design. We hypothesized that a central composite design can be an alternative to factorial arrangements to test a high number of levels for two or more variables and provide similar results.

## Material and Methods

### General

The Kansas State University Institutional Animal Care and Use Committee approved the protocol used in these experiments.

### Animals and diets

Two studies were conducted at a commercial research nursery in southwest Minnesota (Leavenworth Livestock Research Facility, Hubbard Feeds, Sleepy Eye, MN). The rooms were mechanically ventilated with totally slatted floors. Each pen (2.44 × 3.81 m^2^) was equipped with a six-hole stainless steel dry self-feeder (SDI, Alexandria, SD) and a stainless-steel bowl waterer for ad libitum access to feed and water. The first experiment was conducted from August 5 to August 25, 2023, and the second experiment was conducted from July 1 to July 22, 2024. All treatment diets were manufactured at the Hubbard Feeds feed mill in Mankato, MN. In both experiments, diets were corn-SBM-based, and soy hulls and soybean oil were added to change the NE content of the diet. The SBM NE value used in diet formulation was assumed to be 2,405 kcal/kg (90% NE of corn; NRC, 2012). Feed-grade amino acids and SBM were used to adjust SID Lys and SBM content, respectively, and to maintain SID Thr, Trp, Met, Val, and Ile ratios relative to SID Lys (Tables 1 and 2). Daily feed additions to each pen were accomplished using a computerized feeding system (DryExact Pro; Big Dutchman North America, Holland, MI) to record feed deliveries for individual pens. Pens of pigs were weighed at the beginning and at the end of the studies to determine average daily gain (ADG), average daily feed intake (ADFI), and gain-to-feed ratio (G:F).

**Table 1.** Composition of experimental diets, experiment 1 (as-fed basis)^1^^,2^

Item	Diet 1	Diet 2	Diet 3	Diet 4	Diet 5	Diet 6	Diet 7	Diet 8
Ingredients, %								
Corn	56.48	49.15	58.85	51.24	65.18	55.58	66.79	56.87
Soybean meal, 47.7% CP	25.50	35.57	25.50	35.57	25.50	25.50	25.50	35.57
Soybean hulls	13.30	11.58	12.72	10.94	---	---	---	---
Soybean oil	---	---	---	---	4.29	4.87	4.48	5.08
Limestone	0.46	0.47	0.48	0.49	0.65	0.66	0.66	0.64
Monocalcium P, 21% P	0.94	0.86	0.94	0.85	0.95	0.95	0.95	0.86
Salt	0.65	0.65	0.65	0.65	0.65	0.65	0.65	0.65
L-Lys-HCl	0.87	0.56	0.31	---	0.92	0.36	0.36	0.04
DL-Met	0.42	0.32	0.14	0.05	0.42	0.15	0.15	0.06
L-Trp	0.13	0.07	0.03	---	0.13	0.04	0.04	---
L-Thr	0.44	0.30	0.15	0.01	0.46	0.17	0.17	0.03
L-Val	0.33	0.16	0.02	---	0.35	0.03	0.03	---
L-Ile	0.28	0.11	0.01	---	0.30	0.02	0.02	---
Vitamin-trace mineral premix^2^	0.18	0.18	0.18	0.18	0.18	0.18	0.18	0.18
Phytase^3^	0.02	0.02	0.02	0.02	0.02	0.02	0.02	0.02
TOTAL	100	100	100	100	100	100	100	100
Calculated analysis								
Standardized ileal digestible (SID) amino acids								
Lys, %	1.52	1.52	1.08	1.08	1.52	1.52	1.08	1.08
Ile:Lys	60	60	60	74	60	60	60	73
Leu:Lys	87	103	124	146	87	102	124	144
Met:Lys	44	41	37	32	44	41	38	33
Met and Cys:Lys	60	60	60	60	60	60	60	60
Thr:Lys	65	65	65	65	65	65	65	65
Trp:Lys	21	21	21	23	21	21	21	23
Val:Lys	70	70	70	84	70	70	70	83
His:Lys	27	33	39	47	27	32	38	46
Total Lys, %	1.68	1.70	1.24	1.26	1.65	1.67	1.21	1.23
NE, kcal/kg	2,334	2,334	2,334	2,334	2,762	2,762	2,762	2,762
SID Lys:NE, g/Mcal	6.51	6.51	4.63	4.63	5.50	5.50	3.91	3.91
CP, %	19.4	22.7	18.2	21.6	18.5	21.9	17.3	20.8
SID Lys:CP, %	7.84	6.69	5.93	5.00	8.22	6.94	6.24	5.19
Ca, %	0.55	0.57	0.55	0.58	0.55	0.58	0.56	0.58
Available P, %	0.46	0.46	0.46	0.46	0.46	0.46	0.46	0.46

1 Fed from approximately 13 to 25 kg.

2 Vitamin and trace mineral premix with added Alltech Sel-Plex 600 (Alltech, Nicholasville, KY) at the same amount in all the diets.

3 Quantum Blue 5G (AB Vista, Marlborough, UK) was included at 680 FTU/kg, providing an estimated release of 0.16% available P.

**Table 2.** Composition of experimental diets, experiment 2 (as-fed basis)^1^^,2,3^

Item	Diet 1	Diet 2	Diet 3	Diet 4	Diet 5	Diet 6		Diet 7		Diet 8
Ingredients, %										
Corn	60.43	57.77	55.02	52.83	65.16		63.34		57.48	56.03
Soybean meal, 47.7% CP	25.49	25.52	33.53	33.56	25.50		25.48		33.57	33.55
Soybean hulls	9.80	11.00	7.80	8.75	2.30		2.90		2.00	2.40
Soybean oil	1.00	1.05	0.85	0.90	3.60		3.45		4.05	3.90
Limestone	0.70	0.68	0.77	0.75	0.81		0.79		0.85	0.84
Monocalcium P, 21% P	1.03	1.03	1.00	1.00	1.03		1.03		1.00	1.00
Salt	0.65	0.65	0.65	0.65	0.65		0.65		0.65	0.65
L-Lys-HCl	0.33	0.77	0.08	0.52	0.35		0.80		0.10	0.55
DL-Met	0.15	0.36	0.07	0.29	0.15		0.36		0.08	0.30
L-Trp	0.04	0.11	---	0.07	0.05		0.11		---	0.07
L-Thr	0.15	0.38	0.04	0.27	0.15		0.39		0.04	0.28
L-Val	0.05	0.30	---	0.16	0.06		0.31		---	0.17
L-Ile	---	0.22	---	0.08	0.01		0.23		---	0.09
Vitamin-trace mineralpremix^4^	0.18	0.18	0.18	0.18	0.18		0.18		0.18	0.18
Phytase^5^	0.02	0.02	0.02	0.02	0.02		0.02		0.02	0.02
TOTAL	100	100	100	100	100		100		100	100
Calculated analysis										
Standardized ileal digestible (SID) amino acids										
Lys, %	1.08	1.43	1.08	1.43	1.08		1.43		1.08	1.43
Ile:Lys	60	60	73	60	60		60		72	60
Leu:Lys	127	95	145	106	127		95		145	108
Met:Lys	37	42	33	40	37		42		33	40
Met and Cys:Lys	60	60	60	60	60		60		60	60
Thr:Lys	65	65	65	65	65		65		65	65
Trp:Lys	21	21	21	21	21		21		21	21
Val:Lys	71	70	78	70	70		70		78	70
His:Lys	40	30	47	35	40		30		47	35
Total Lys, %	1.23	1.58	1.25	1.59	1.21		1.56		1.23	1.58
NE, kcal/kg	2,425	2,425	2,425	2,425	2,676		2,676		2,676	2,676
SID Lys:NE, g/Mcal	4.45	5.88	4.45	5.89	4.03		5.34		4.03	5.34
CP, %	18.7	19.7	21.5	22.3	18.4		19.4		21.1	22.0
SID Lys:CP, %	5.78	7.26	5.02	6.41	5.87		7.37		5.12	6.50
Ca, %	0.62	0.61	0.65	0.65	0.62		0.61		0.65	0.65
Available P, %	0.46	0.46	0.46	0.46	0.46		0.46		0.46	0.46

1 Fed from approximately 10 to 23 kg.

2 Diets one to eight were fed as is to accomplish the nutrient requirements for treatments 1, 4, 5, 8, 9, 12, 13, and 16, respectively.

3 The following treatments were accomplish by the following blends: treatment 2 (Diet 1 [75%] and 4 [25%]); treatment 3 (Diet 1 [25%] and 4 [75%]); treatment 6 (Diet 5 [75%] and 8 [25%]); treatment 7 (Diet 5 [25%] and 8 [75%]); treatment 10 (Diet 9 [75%] and 12 [25%]); treatment 11 (Diet 9 [25%] and 12 [75%]); treatment 14 (Diet 13 [75%] and 16 [25%]); treatment 15 (Diet 13 [25%] and 16 [75%]).

4 Vitamin and trace mineral premix with added Alltech Sel-Plex 600 (Alltech, Nicholasville, KY) at the same amount in all the diets.

5 Quantum Blue 5G (AB Vista, Marlborough, UK) was included at 680 FTU/kg, providing an estimated release of 0.16% available P.

### Experiment 1—Central composite design

A total of 4,681 pigs (PIC 337 × 1,050; initially 13.0 ± 0.36 kg) were used in a 21-d trial to investigate the interactive effects of NE, SBM, and SID Lys content on growth performance of nursery pigs. Pigs were housed in mixed-sex pens with approximately 35 pigs per pen and assigned in a randomized complete block design to 15 dietary treatments using a circumscribed CCD. The experimental design consisted of seven blocks, each comprising 19 pens: eight factorial points (treatments 1 to 8), six axial points (treatments 9 to 14), and a central point (treatment 15) replicated five times. By design, the central point is replicated at least three times more than each of the factorial and axial point treatments. This resulted in seven replications for each factorial and axial points, and 35 replications for the central points, totaling 133 pens across the study. Eight diets were formulated with various levels of NE, SID Lys, and SBM (Table 1) and blended to create the 15 dietary treatments (Table 3). The NE ranged from 2,334 to 2,762 kcal/kg, SBM from 25.5% to 35.9%, and SID Lys from 1.08% to 1.52%.

**Table 3.** Nutrient composition and diet blend proportion used to meet the nutrient levels of the fifteen dietary treatments of experiment 1

	Nutrient level			Diet blend, %^1^							
Treatment	NE, kcal/kg	SBM, %	SID Lys, %	1	2	3	4	5	6	7	8
1	2,421	27.6	1.17	---	---	80	---	---	20	---	---
2	2,676	27.6	1.17	---	20	---	---	---	---	80	---
3	2,421	27.6	1.43	80	---	---	---	---	---	---	20
4	2,676	27.6	1.43	---	---	---	20	80	---	---	---
5	2,421	33.8	1.17	---	---	---	80	20	---	---	---
6	2,676	33.8	1.17	20	---	---	---	---	---	---	80
7	2,421	33.8	1.43	---	80	---	---	---	---	20	---
8	2,676	33.8	1.43	---	---	20	---	---	80	---	---
9	2,334	30.7	1.30	---	50	50	---	---	---	---	---
10	2,763	30.7	1.30	---	---	---	---	---	50	50	---
11	2,549	30.7	1.08	---	---	50	---	---	---	---	50
12	2,549	30.7	1.52	50	---	---	---	---	50	---	---
13	2,549	25.5	1.30	50	---	---	---	---	---	50	---
14	2,549	35.9	1.30	---	---	---	50	---	50	---	---
15	2,549	30.7	1.30	---	---	50	---	---	50	---	---

1 Percentage of the eight diets manufactured to meet the requirements of the 15 dietary treatments.

### Experiment 2—Factorial arrangement

A total of 4,336 pigs (PIC 337 × 1,050; initially 10.6 ± 0.32 kg) were used in a 21-d trial to investigate the interactive effects of NE, SBM, and SID Lys content on the growth performance of pigs from 11 to 23 kg. Pigs were housed in mixed-sex pens with approximately 34 pigs per pen, with eight replications (blocks) per treatment. Dietary treatments were arranged in a 2 × 2 × 4 factorial with 16 dietary treatments. Main effects consisted of NE (2,425 or 2,676 kcal/kg), SBM (25.5% or 33.5% of the diet), and SID Lys (1.08%, 1.20%, 1.31%, or 1.43%). Eight diets were formulated to various NE, SBM, and SID Lys concentrations and blended to create the 16 dietary treatments (Table 2).

### Chemical analysis

Feed samples were collected from multiple feeders, blended, subsampled, ground, and analyzed for dry matter, crude protein, crude fiber, ether extract, and ash content (experiment 1: Midwest Laboratories, Inc., Omaha, NE; experiment 2: University of Missouri Agricultural Experiment Station Chemical Laboratory, Columbia, MO), and total amino acids profile (experiment 1: Ajinomoto Health & Nutrition North America, Inc., Eddyville, IA; experiment 2: University of Missouri Agricultural Experiment Station Chemical Laboratory, Columbia, MO).

### Statistical analysis

In both experiments, data were analyzed using R [version 4.1.1 (August 10, 2021), R Foundation for Statistical Computing, Vienna, Austria] with pen considered the experimental unit, treatment as a fixed effect, and initial weight per pen as the blocking factor. In experiment 1, data were analyzed as a central composite design of response surface methodology using the RSM function from the RSM package in R software and visualized using three-dimension surface plots. The adjusted R^2^ reported indicates the amount of variation in the response explained by the predictor variables. Lack of fit measures the discrepancy between the observed data and the predicted value estimated by the model, meaning that if significant the model does not capture the relationship adequately. Pure error reflects the variation in the response variable that is due to the natural variability in the experimental process. Some of the variables showed a strong relationship analyzed in the confidence interval between 95% and 90%; therefore, all results were considered significant at *P *≤ 0.10.

In experiment 2, data were analyzed as a randomized complete block design using the lmer function from the lme4 package in R software. Three-way and two-way interactions, and the main effect of NE, SBM, and SID Lys were analyzed, and the highest order model was reported when statistical significance was observed. Polynomial contrast coefficients were used to compare the effect of increasing levels of SID Lys. Differences between treatments were considered significant at *P *≤ 0.05 and marginally significant at *P > *0.05 and <0.10.

### Model validation

The predictor variables obtained in experiment 1 (Table 7) were used to predict ADG and G:F using the concentrations of NE, SBM, and SID Lys in experiment 2 (factorial arrangement). The observed and predicted responses were compared to determine the accuracy of the predicted responses following the procedures by Kerkaert et al. (2021). Treatment 7 from experiment 1 and treatment 8 from experiment 2 had the same ingredient composition. Therefore, the intercept term in the equation from the predicted value of treatment 8 was adjusted until the predicted ADG, ADFI, and G:F matched the observed value for this treatment in experiment 2. Then, the adjusted intercept was used to predict ADG, ADFI, and G:F for the remaining treatments. These values were then compared to the observed values.

**Table 7.** Regression equations for prediction of nursery pig ADG, ADFI, and G:F in response to net energy (NE), SID lysine (Lys), and soybean meal (SBM) content

Response variable	Regression equation^1^	*P*	Adjusted R^2^
ADG, kg	=0.579392 − (0.0063389 × NE) + (0.0067428 × SBM) + (0.0260796 × Lys) − (0.0065721 × Lys²)	<0.001	0.355
ADFI, kg	=0.8210353 − (0.0136864 × NE) + (0.0024825 × SBM) + (0.0066549 × Lys)	0.040	0.040
G:F, g/kg	=0.7013215 + (0.0035198 × NE) + (0.0060863 × SBM) + (0.0264389 × Lys) − (0.0048012 × NE²) - (0.0031838 × Lys²) − (0.0034413 × Lys × SBM)	<0.001	0.654

1 Equations from central composite design analysis are expressed relative to user input. Thus, dietary components in the equations need to be included as follows: NE= [User NE (kcal/kg) – 2,548.5] ÷ 214; SBM = [User SBM (%) – 30.7] ÷ 5.2; SID Lys = [User SID Lys (%) – 1.3] ÷ 0.22

## Results

### General

Overall, pigs in both experiments were healthy, and there were minimal removals due to injury, health, or poor performance. In both experiments, chemical analysis of complete diets was consistent with the calculated values used in diet formulation (Tables 4 and 5).

**Table 4.** Chemical analysis of experiment 1 diets (as fed-basis)^1^

	Item		
Diet	CP, %	Ether extract, %	Total Lys, %
1	17.8	3.16	1.36
2	19.7	5.57	1.28
3	19.0	3.41	1.53
4	18.9	5.52	1.57
5	19.6	3.14	1.29
6	20.8	6.13	1.37
7	20.0	3.40	1.53
8	20.7	6.06	1.51
9	18.6	2.36	1.43
10	18.1	7.04	1.38
11	20.6	5.21	1.24
12	19.9	5.35	1.64
13	17.8	4.96	1.38
14	19.9	4.62	1.35
15	19.8	4.63	1.40

1 Values represent means from four composite samples. For each treatment, samples were collected from multiple feeders, blended, subsampled, ground, and analyzed (Midwest Laboratories, Inc., Omaha, NE; Ajinomoto Health & Nutrition North America, Inc., Eddyville, IA).

**Table 5.** Chemical analysis of experiment 2 diets (as fed-basis)^1^

	Item		
Diet	CP, %	Ether extract, %	Total Lys, %
1	17.4	2.5	1.30
2	18.3	2.7	1.44
3	18.4	2.7	1.53
4	19.2	2.9	1.60
5	20.9	2.2	1.31
6	20.7	2.4	1.48
7	20.8	2.5	1.60
8	21.1	2.5	1.68
9	17.6	5.4	1.33
10	18.3	5.8	1.44
11	18.1	5.7	1.43
12	18.6	6.2	1.65
13	19.5	4.0	1.23
14	20.3	3.5	1.48
15	20.2	3.7	1.48
16	21.3	4.3	1.64

1 Values represent means from four composite samples. For each treatment, samples were collected from multiple feeders, blended, subsampled, ground, and analyzed (University of Missouri Agricultural Experiment Station Chemical Laboratory, Columbia, MO).

### Experiment 1—Central composite design

**Figure 1.**
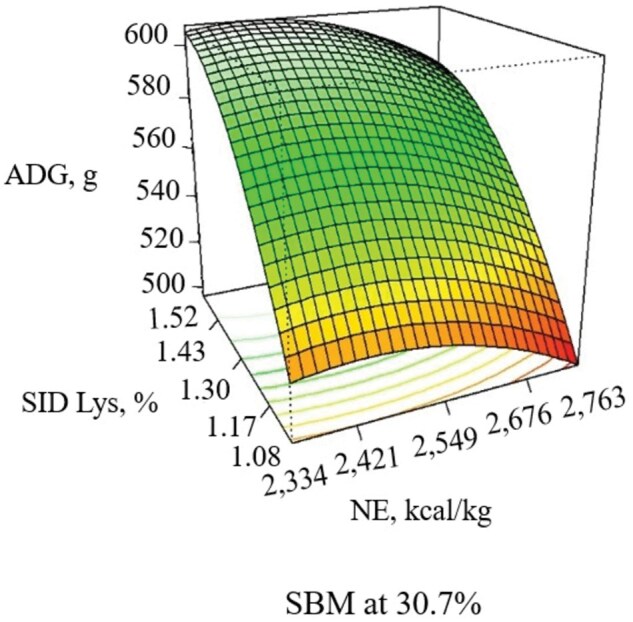
Response surface plot of NE (Linear, *P *= 0.052) and SID Lys (Quadratic, *P *= 0.044) on ADG with SBM held at the midpoint (30.7%) in pigs from 11 to 25 kg. Adj. R^2^= 0.355

**Figure 2.**
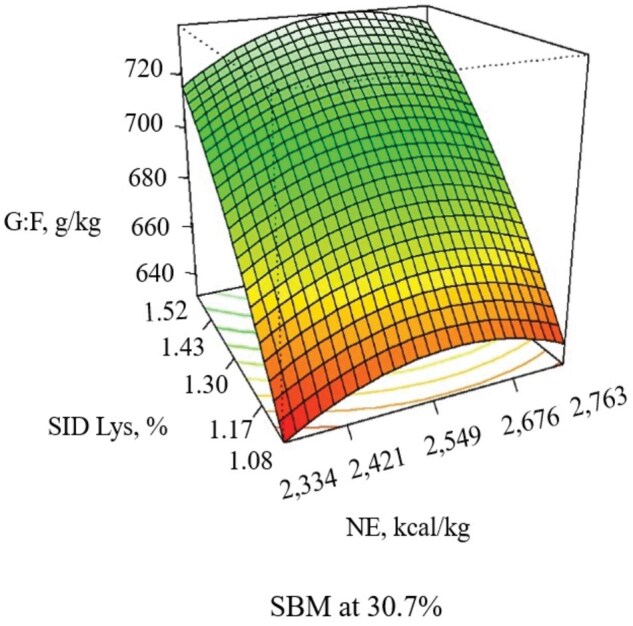
Response surface plot of NE (Quadratic, *P *= 0.005) and SID Lys (Lys × SBM, *P *= 0.082) on G:F with SBM held at the midpoint (30.7%) in pigs from 11 to 25 kg. Adj. R^2^= 0.655

**Figure 3.**
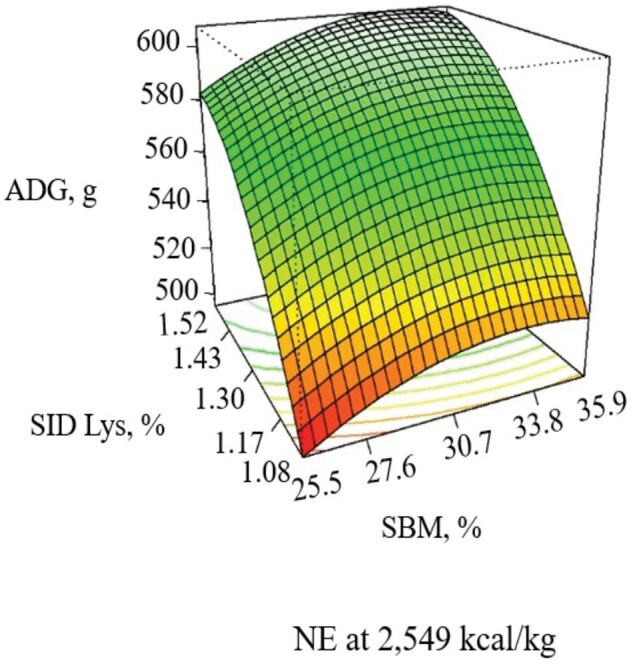
Response surface plot of SBM (Linear, *P *= 0.039) and SID Lys (Quadratic, *P *< 0.044) on ADG with NE held at the midpoint (2,549 kcal/kg) in pigs from 11 to 25 kg. Adj. R^2^= 0.355

**Figure 4.**
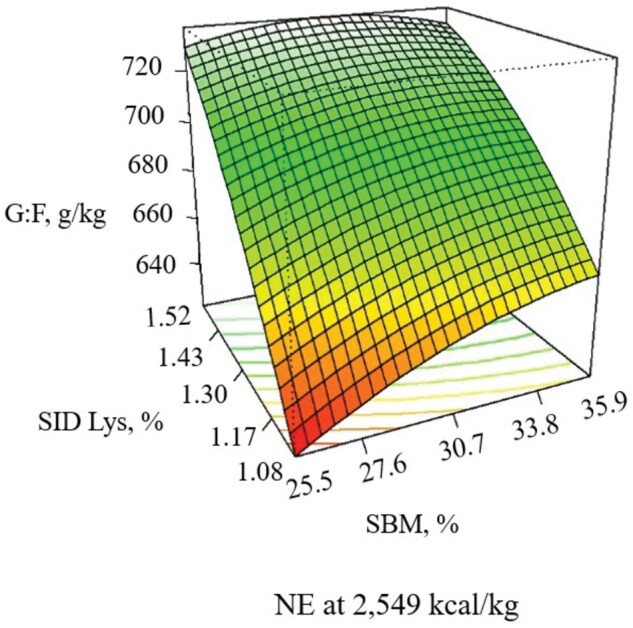
Response surface plot of SBM (Lys × SBM, *P *= 0.082) and SID Lys (Lys × SBM, *P *= 0.082) on G:F with NE held at the midpoint (2,549 kcal/kg) in pigs from 11 to 25 kg. Adj. R^2^ = 0.655

During the 21-d experimental period, increasing NE decreased ADG (linear, *P *= 0.052), and ADFI (linear, *P *= 0.011), with the response in G:F being quadratic (*P *= 0.005), with G:F increasing as NE increased until approximately 2,600 kcal/kg (Table 6; Figures 1 and 2). Increasing SBM increased ADG (linear, *P *= 0.039) and G:F (linear, *P *< 0.001) when included at 33.8% of the diet without additional benefits thereafter (Figure 3). When increasing SID Lys, ADG (quadratic, *P *= 0.044) and G:F (quadratic, *P *= 0.020) increased to approximately 1.43% with little benefit thereafter (Figure 3). There was an interaction (*P *= 0.082) observed between SID Lys and SBM on G:F, where increasing SBM in diets with low SID Lys provided a greater response in G:F than when SBM was increased in diets with high SID Lys levels (Figure 4). The CCD results provided linear and quadratic coefficients to develop models to predict ADG, ADFI, and G:F using the significant terms of NE, SBM, and SID Lys as predictor variables (Table 7).

**Table 6.** Performance of nursery pigs fed five levels of net energy (NE), standardized ileal digestible lysine (SID Lys), and soybean meal (SBM) content using a central composite design (experiment 1)^1^

	Nutrient level^2^			Growth performance response			
Treatment	NE^3^	SBM^4^	Lys^5^	Final BW, kg	ADG, g	ADFI, g	G:F, g/kg
1	2,420	27.6	1.17	23.6	542	828	654
2	2,677	27.6	1.17	23.0	516	789	653
3	2,420	27.6	1.43	24.4	585	819	715
4	2,677	27.6	1.43	24.4	579	806	719
5	2,420	33.8	1.17	23.9	547	822	666
6	2,677	33.8	1.17	23.6	534	783	682
7	2,420	33.8	1.43	24.8	594	831	714
8	2,677	33.8	1.43	25.1	604	827	731
9	2,334	30.7	1.30	24.7	588	863	682
10	2,762	30.7	1.30	24.1	558	809	689
11	2,549	30.7	1.08	23.5	526	809	650
12	2,549	30.7	1.52	24.7	604	827	731
13	2,549	25.5	1.30	23.9	562	823	683
14	2,549	35.9	1.30	24.7	583	831	701
15	2,549	30.7	1.30	24.4	579	826	701
SEM				0.77	12.2	20.2	6.6
R^2^				0.059	0.385	0.073	0.670
Adjusted R^2^				0.014	0.355	0.029	0.655
*P* ^6^							
Interactions							
NE × Lys				NS	NS	NS	NS
NE × SBM				NS	NS	NS	NS
SBM × Lys				NS	NS	NS	0.082
Quadratic							
NE				NS	NS	NS	0.005
SBM				NS	NS	NS	NS
Lys				NS	0.044	NS	0.020
Linear							
NE				0.546	0.052	0.011	0.047
SBM				0.241	0.039	0.639	<0.001
Lys				0.017	< 0.001	0.210	<0.001
Lack of fit^7^				0.999	0.798	0.923	0.822
Pure error^8^				4.41	0.07	0.12	0.04

1 A total of 4,681 pigs (PIC 337 × 1,050, initially 13.0 ± 0.36 kg) were used in a 21-d experiment using a central composite design of response surface methodology. Approximately 35 pigs per pen were used and assigned to 1 of 15 treatments in a randomized complete block design. Within each block, factorial points and axial points were replicated once, and the central point was replic**ated** five times for a total of 19 pens per block. Means represent the average of seven replications per treatment, except treatment 15, which is replicated 35 times.

2 Each of the three factors NE, SID Lys, and SBM content was tested in five different levels (−α, −1, 0, +1, and  + α) calculated based on the central values (0) selected for this period.

3 Net energy levels (kcal/kg): −α = 2,334; −1 = 2,420; 0 = 2,549; +1 = 2,677; and  + α = 2,762.

4 Soybean meal (%): −α = 25.5; −1 = 27.6; 0 = 30.7; +1 = 33.8; and  + α = 35.9.

5 SID Lys levels (%): −α = 1.08; −1 = 1.17; 0 = 1.30; +1 = 1.43; and  + α = 1.52.

6 Results were considered significant at a *P* value ≤ 0.10. NS = Non-significant.

7 Indicates how well the model fits the data. The higher the lack of fit *P*-value indicates the better fitting model for the responses analyzed.

8 Standard deviation of replicates at the center points. Calculated as √MSE.

### Experiment 2—Factorial arrangement

**Figure 5.**
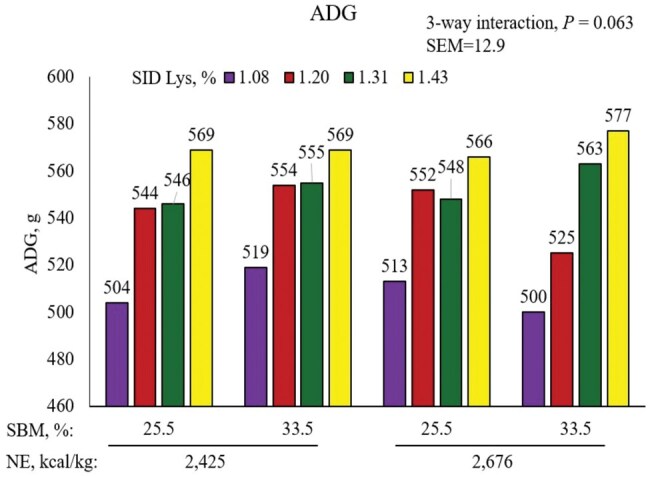
Interactive effects of NE, SBM, and SID Lys on ADG of pigs from 11 to 23 kg.

There was a tendency for a three-way interaction (*P *= 0.063) in ADG between NE, SBM, and SID Lys (Figure 5; Table 8). Average daily gain increased as SID Lys increased in diets containing 2,627 kcal NE/kg and 33.5% SBM, whereas the response to SID Lys appeared to be quadratic for pigs fed 2,425 kcal NE or 25.5% SBM with 2,676 kcal NE. The main effect of SID Lys was the only significant (linear, *P *< 0.001) main effect response for ADG, with ADG increasing as SID Lys increased.

**Table 8.** Interactive and main effects of feeding pigs two levels of net energy (NE) and soybean meal (SBM) with four levels of SID lysine (Lys) on growth performance (experiment 2)^1^

Nutrient level				Growth performance response			
Treatment	NE^2^	SBM^3^	Lys^4^	BW, kg	ADG, g	ADFI, g	G:F, g/kg
1	2,425	25.5	1.08	21.3	504	764	660
2	2,425	25.5	1.20	22.0	544	784	695
3	2,425	25.5	1.31	22.1	546	771	709
4	2,425	25.5	1.43	22.6	569	786	724
5	2,425	33.5	1.08	21.6	519	749	694
6	2,425	33.5	1.20	22.3	554	768	722
7	2,425	33.5	1.31	22.3	555	758	732
8	2,425	33.5	1.43	22.6	569	771	739
9	2,676	25.5	1.08	21.4	513	757	678
10	2,676	25.5	1.20	22.2	552	773	714
11	2,676	25.5	1.31	22.1	548	751	730
12	2,676	25.5	1.43	22.5	566	757	748
13	2,676	33.5	1.08	21.1	500	735	680
14	2,676	33.5	1.20	21.9	525	741	709
15	2,676	33.5	1.31	22.5	563	768	733
16	2,676	33.5	1.43	22.8	577	757	763
SEM				0.52	12.9	19.2	6.8
*P* ^5^							
3-way interaction^6^							
Linear NE × SBM × SID Lys				0.174	0.063	0.298	0.036
2-way interactions^6^							
NE × SBM				0.604	0.230	0.645	<0.001
Linear NE × SID Lys				0.495	0.360	0.718	0.006
Linear SBM × SID Lys				0.529	0.419	0.250	0.724
Main effects							
NE				0.766	0.651	0.018	0.001
SBM				0.499	0.584	0.043	<0.001
SID Lys, linear				<0.001	<0.001	0.085	<0.001
SID Lys, quadratic				0.172	0.068	0.407	0.015

1 A total of 4,336 pigs (PIC 337 × 1,050, initially 10.6 ± 0.32 kg) were used in a 21-d experiment. Approximately 34 pigs per pen were used and assigned to 1 of 16 treatments in a randomized complete block design.

2 Net energy (kcal/kg).

3 Soybean meal (%).

4 SID Lys (%).

5 Results were considered significant at *P *≤ 0.05 and marginally significant at 0.05 < *P *< 0.10. NS= non-significant.

6 Quadratic terms of SID Lys with the two other factors were not significant (*P *> 0.10).

There was a main effect of NE (*P *= 0.018) and SBM (*P *= 0.043) observed for ADFI. High NE and SBM levels decreased ADFI compared with low NE and SBM. There was also a tendency (linear, *P *= 0.085) for increased ADFI as SID Lys increased.

**Figure 6.**
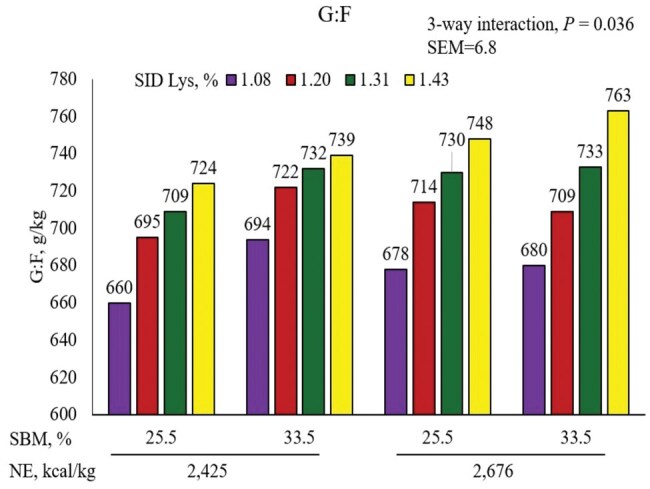
Interactive effects of NE, SBM, and SID Lys on G:F of pigs from 11 to 23 kg.

A three-way interaction (linear, *P *= 0.063) was observed between NE, SBM, and SID Lys for G:F (Figure 6). Increasing SBM from 25.5% to 33.5% increased G:F to a greater extent in low-energy diets than the high-energy diets. Increasing SID Lys provided a greater response in the high-energy diets than in the low-energy diets. The increased G:F in the diet containing 1.08% SID Lys when NE was at 2,425 kcal/kg and SBM at 33.5% as compared to when the same SID Lys and NE were fed with low SBM also contributed to the three-way interaction.

### Model validation

The ADG and G:F predictions, obtained using predictor variables from experiment 1, were within ± 3% of the observed values in experiment 2 for all variables and treatments except for treatment 10 (2,676 kcal/kg NE, 25.5% SBM, and 1.20% SID Lys), where ADG and ADFI were underpredicted by 5% and 4%, respectively (Table 9).

**Table 9.** Predicted values of average daily gain (ADG), average daily feed intake (ADFI), and gain-to-feed ratio (G:F) using the nutrient levels from experiment 2 with the estimators obtained in experiment 1

	Observed^1^			Predicted^2^			%^3^		
Treatment	ADG	ADFI	G:F	ADG	ADFI	G:F	ADG	ADFI	G:F
1	504	764	660	513	757	683	98	101	97
2	544	784	695	532	760	702	102	103	99
3	546	771	709	547	764	718	100	101	99
4	569	786	724	559	767	733	102	102	99
5	519	749	694	523	760	698	99	99	99
6	554	768	722	543	764	714	102	101	101
7	555	758	732	557	767	727	100	99	101
8	569	771	739	569	771	739	100	100	100
9	513	757	678	505	741	687	102	102	99
10	552	773	714	525	744	706	105	104	101
11	548	751	730	539	747	722	102	101	101
12	566	757	748	551	751	737	103	101	101
13	500	735	680	516	744	702	97	99	97
14	525	741	709	535	748	718	98	99	99
15	563	768	733	550	751	731	102	102	100
16	577	757	763	562	755	743	103	100	103

1 Mean values observed in experiment 2

2 Values were obtained using the estimators in Table 6 and the nutrient levels of experiment 2. Treatments 7 and 8 from experiment 1 and 2, respectively, had the same ingredient composition. Therefore, the intercept term in the equation from the predicted value of treatment 8 was adjusted until the predicted ADG and G:F matched the observed value in experiment 2. Then, the adjusted term of each response was used to predict the remaining treatments.

3 (Observed ÷ Predicted) × 100.

## Discussion

Robinson and Nielson (1960) discouraged the use of a CCD with three independent variables because of the risk of high variability. However, CCDs have been widely used in poultry nutrition research, but not commonly used in swine nutrition until recently (Humphrey et al., 2023). Central composite design provides first and second-order models and interactions of a large number of variables and their levels with a reduced number of treatments compared to a full factorial model. Response surface designs like CCDs are used to determine, within some limits, the optimum operating conditions of a system (Box and Wilson, 1951). Factorial arrangements for their part are used to assess main effects and interactions between factors, but not curvature. The CCD is more efficient when a second-order surface is adequate for representing the data and locating optimum conditions, but the feasibility of applying treatment combinations at the axial points levels needs to be addressed. The factorial and central points used in CCD estimate the interaction and main effects between variables. The axial points are the extreme values that augment the research area, providing the curvature of the responses. The values obtained at the different points are then used to estimate the intermediate points that were not actually tested, thereby minimizing the number of treatments with a CCD. Although fewer treatments are needed compared to a factorial arrangement, a CCD still requires a high number of treatments and pens (experimental units) (Beg and Rahman, 2021), which is the primary limiting factor for expanding its use in swine research. The main objective of this paper was to validate the usefulness of CCD by comparing the predicted responses with the results obtained with a factorial arrangement. To this end, experiment 2 was conducted in a factorial arrangement with main effects and diets formulated to approximately the same ranges of SBM (25.5% to 33.5%), NE (2,425 to 2,676 kcal/kg), and SID Lys (1.08% to 1.43%) similar to those used in experiment 1.

In both experiments, the ADG response was affected by NE, SBM, and SID Lys, where SID Lys and its ratio to NE had a large influence on the results in both experiments. The response to SBM was more subtle, with a smaller linear main effect in experiment 1 and as part of a three-way interaction in experiment 2.

Increasing SID Lys increased ADG in a quadratic and linear manner in experiment 1 and 2, respectively. In experiment 1, no further improvements were observed above 1.43% SID Lys. Menegat et al. (2019) observed that the ideal SID Lys:NE ratio for an 11 to 25 kg pig is approximately 5.27 g/Mcal NE. In experiment 1, the diets containing 1.43% SID Lys were above the ideal SID Lys:NE ratio (5.61 g/Mcal); therefore, no further improvements in ADG were observed when SID Lys was above 1.43%. In experiment 2, the highest level of SID Lys used at both NE concentrations was within an ideal Lys:NE ratio for this weight range; therefore, it showed a linear response as SID Lys increased. The linear increase in ADG as SID Lys increased in the diet was also observed in other studies when SID Lys increased using a fixed NE level on pigs over 13 kg of BW (Aymerich et al., 2020; Xue et al., 2022).

As previously mentioned, the ideal Lys:NE ratio for an 11 to 25 kg pig is approximately 5.27 g/Mcal NE. Therefore, increasing NE in the diet has to be followed by increasing SID Lys to optimize performance (Marçal et al., 2019). The reduction in ADG observed was due to a reduction in the SID Lys:NE ratio as NE increased in experiment 1. This is because a CCD does not test all possible treatment combinations, and most of the high NE concentrations tested were under the ideal Lys:NE ratio. In experiment 2, at high NE (2,676 kcal/kg) concentrations, the four SID Lys levels tested resulted in a wider range of Lys:NE ratios compared to the low NE (2,425 kcal/kg) treatments. Again, the treatment containing 1.43% SID Lys was at an ideal SID Lys:NE ratio, but the ratio reduced with lower SID Lys, resulting in a linear decrease in ADG. A recent meta-analysis by Goethals et al. (2024) reported that pigs between 11 and 25 kg require from 5.42 to 5.83 g of SID Lys per Mcal of NE to maximize growth performance. This supports that the ratio between dietary NE and SID Lys that should be maintained to support optimum growth performance; however is not always observed at this stage of growth (Lunedo et al., 2023). Pigs typically adjust their feed intake to meet their energy requirement, and that is why the SID Lys levels must be adjusted to avoid Lys deficiencies if high-energy diets are fed (Chiba et al., 1991).

In experiment 1, increasing SBM resulted in a linear increase in ADG; however, no responses to increasing SBM were observed in experiment 2. Cemin et al. (2020a) evaluated increasing SBM (27.5% to 37.5% of the diet) in 11 to 25 kg pigs and observed inconsistent ADG responses, with no evidence for differences in three studies, but a linear decrease in ADG as SBM increased in one of the experiments. The small increase in ADG as SBM increased is possibly due to the reduction in the Lys:CP ratio, as SBM provided more non-essential amino acids and N to the diet. The improvements observed in ADG also could be caused by the bioactive compounds in the SBM. A review by White et al. (2024) reported that SBM bioactive compounds, such as oligosaccharides and isoflavones, may play a beneficial role in the performance of the pigs. The antioxidant effect of isoflavones in pigs is well documented, with the reasons for the beneficial effect of oligosaccharides less clear, but the influence on pig growth performance could be related to the level of these compounds increasing as SBM is increased in the diet.

In both experiments, ADFI decreased as dietary NE increased. Even though Lunedo et al. (2023) indicated that increasing NE did not affect ADFI of pigs from 7 to 20 kg of BW, other studies have indicated that feed intake decreases with increasing NE in 11 to 15 kg pigs (Beaulieu et al., 2006; Mendoza and Van Heugten, 2014). This indicates that pigs will adjust their intake as dietary NE increases to more closely reach their daily caloric requirement (Chiba et al., 1991).

Feed intake was decreased when feeding 33.5% SBM compared to 25.5% in experiment 2, but there were no differences observed in experiment 1. Cemin et al. (2020a) observed a linear reduction in ADFI as SBM increased from 27.5% to 37.5% in two of four studies. Faccin et al. (2023) also observed a linear reduction in ADFI in two studies when feeding 25% to 40% SBM to 11 to 28 kg pigs. The results herein, as well as those of Cemin et al. (2020a) and Faccin et al. (2023), might suggest that very high levels of SBM in the diet decrease ADFI, possibly due to palatability. Heo et al. (2013) suggested that high CP levels fed in nursery diets increased the fermentation of protein substrates in the large intestine which can result with the production of toxic compounds such as ammonia and amines. The reasons why the reduction in ADFI was observed in experiment 2 and not in experiment 1 are unclear, but it could be attributed to a reduction in palatability and/or the amount of bioactive compounds present as SBM increased.

Increasing SID Lys, linearly increased ADFI. Kendall et al. (2008) conducted five studies evaluating SID Lys from 1.05% to 1.50% of the diet in pigs from 11 to 27 kg and did not observe differences in ADFI as SID Lys increased. This may suggest that the ADFI response observed in our experiments is not related to increasing SID Lys but to other components of the diets.

In experiment 1, an interaction between SID Lys and SBM was observed for G:F, with G:F increasing to a greater extent when SBM levels increased in the diet with low SID Lys than in diets with high SID Lys. This response is possibly caused by the other essential amino acids, non-essential amino acids, and N provided as SBM content increased in the diet (Pope et al., 2023). In experiment 2, there was a 3-way interaction between the NE, SBM, and SID Lys levels, driven by increased G:F in the diet containing 1.08% SID Lys when NE was at 2,425 kcal/kg and SBM at 33.5% as compared to when the same SID Lys and NE were fed with low SBM. Most of the essential amino acids are commercially available to be added in a feed-grade form in the diet; however, the non-essential amino acids can become limiting when the SID Lys to crude protein content becomes too high to provide adequate N for non-essential amino acid synthesis. Additionally, although diets were formulated to be above the His:Lys ratio suggested by Cemin et al. (2018), SID His:Lys and other essential amino acids increased as SBM was added to the diets. Therefore, when extra amino acids and protein from SBM were provided, the benefits in pig performance were observed. Similar effects were observed when increasing the CP level of the diet fed to nursery pigs (Kim et al., 2011; Limbach et al., 2021; Correia et al., 2023). Also, Silva et al. (2020) observed greater N retention in carcasses and viscera when more N and non-essential amino acids are fed.

The differences in Lys:NE ratios also may explain part of the three-way interaction observed in experiment 2. When comparing the treatments at the same Lys:NE ratio (2,425 kcal/kg NE), the treatment containing high SBM provided more non-essential amino acids, resulting in greater G:F compared to the pigs fed diets with lower SBM. The same improvements in G:F were not found as SBM increased in the high NE diets (2,676 kcal/kg). The reason is not totally clear; however, these diets would have had lower SID Lys:NE ratios than pigs fed the low NE diets because diets were formulated to a constant SID Lys percentage. Another possibility to consider is that the NE value used for SBM in our diet formulation was 2,405 kcal/kg, equivalent to 90% of the NE value of corn (NRC, 2012). Based on caloric efficiency and digestibility studies, it was observed that the NE content of SBM is greater than the current values estimated by NRC (2012), Rojas and Stein (2013), Li et al. (2017), Cemin et al. (2020b). Thus, the response observed in G:F could be partially attributed to more NE contributed to the diet by SBM.

The validation of the model generated from the CCD showed its ability to predict the responses obtained with the factorial arrangement within ± 3% of the observed values in almost all comparisons. The predictions of treatment 10 were an exception, where the model underpredicted ADG and ADFI by 5% and 4%, respectively. The reason for the discrepancy is not entirely known but appears to be because the negative effect of dietary NE on feed intake was less in the values observed in experiment 2 than the predicted ones, with the greatest discrepancy in treatment 10. Nevertheless, these results demonstrated that CCD can be used to accurately predict changes in growth performance of pigs in response to different dietary components. Therefore, if facility constraints allow for adequate replications, CCD allows a high number of variables and levels to be tested simultaneously.

In summary, the model obtained with the CCD provided accurate estimates for ADG and G:F when applied to similar nutrient levels from the factorial arrangement. In addition, the growth performance results demonstrated the importance of maintaining Lys:NE ratios to improve performance, and the impact of N and other non-essential amino acids (Lys:CP ratio) contributed by SBM to increase G:F. We therefore conclude that CCD can be effectively used in swine research to predict growth performance responses to different nutrient levels.

## Abbreviations:

ADFI: average daily feed intake
ADG: average daily gain
BW: body weight
CP: crude protein
G:F: gain-to-feed ratio
NE: net energy
SID: standardized ileal digestible
SBM: soybean meal
